# Hepatocytes Polyploidization and Cell Cycle Control in Liver Physiopathology

**DOI:** 10.1155/2012/282430

**Published:** 2012-10-22

**Authors:** Géraldine Gentric, Chantal Desdouets, Séverine Celton-Morizur

**Affiliations:** ^1^Division of Cell Cycle, Regeneration and Liver Diseases, EMC Department, Institut Cochin, INSERM U1016, 24 rue du Faubourg Saint Jacques, 75014 Paris, France; ^2^CNRS, UMR8104, Paris, France; ^3^Université Paris Descartes, Sorbonne Paris Cité, Paris, France

## Abstract

Most cells in mammalian tissues usually contain a diploid complement of chromosomes. However, numerous studies have demonstrated a major role of “diploid-polyploid conversion” during physiopathological processes in several tissues. In the liver parenchyma, progressive polyploidization of hepatocytes takes place during postnatal growth. Indeed, at the suckling-weaning transition, cytokinesis failure events induce the genesis of binucleated tetraploid liver cells. Insulin signalling, through regulation of the PI3K/Akt signalling pathway, is essential in the establishment of liver tetraploidization by controlling cytoskeletal organisation and consequently mitosis progression. Liver cell polyploidy is generally considered to indicate terminal differentiation and senescence, and both lead to a progressive loss of cell pluripotency associated to a markedly decreased replication capacity. Although adult liver is a quiescent organ, it retains a capacity to proliferate and to modulate its ploidy in response to various stimuli or aggression (partial hepatectomy, metabolic overload (i.e., high copper and iron hepatic levels), oxidative stress, toxic insult, and chronic hepatitis etc.). Here we review the mechanisms and functional consequences of hepatocytes polyploidization during normal and pathological liver growth.

## 1. Introduction

Polyploidy, the state of having an increase in the number of chromosomes sets, is a widespread physiological phenomenon observed particularly in plants, fungi, insects, fishes, and amphibians [1]. The additional set (or sets) of chromosomes may originate from the same individual (“autopolyploid”) or from the hybridization of two different species (“allopolyploid”). Although diploid is the normal status for mammalian cells, various studies have demonstrated during last decades a major role of “diploid-polyploid conversion” during physiopathological processes in several tissues. Indeed, polyploidy seems to be part of a developmental program resulting in the formation of highly differentiated cells, as it has been reported for megacaryocytes (16n–128n) [2], cardiomyocytes (4n) [3], trophoblast giant cells (8n–64n) [4], Purkinje neurons, [5] and retinal ganglion cells (both 4n) [6] or hepatocytes (4n–8n) in the liver parenchyma [7]. Furthermore, in response to stress or injury, genesis of polyploid contingent can be also observed. Uterine smooth muscle during pregnancy [8], heart muscle and vascular smooth muscle cells (VSMC) during hypertension [9, 10], and thyroid cells in hyperthyroidism [11] are prone to switch to polyploid state. Finally, genesis of polyploid cells by unscheduled whole-genome duplications can also participate to carcinogenesis process, by inducing establishment of chromosomal instability (CIN). Indeed, in many human carcinomas (breast, lung, colon, pancreas, oesophagus) emergence of tetraploid cells has been observed in early steps of tumorigenesis and precede the genesis of cells with intermediate DNA content values (aneuploid cells) [12, 13]. 

Several mechanisms have been involved in the physiopathological emergence of polyploid cells in mammals. During *cell-cell fusion*, genesis of polyploid cells may occur independently of cell proliferation, as it has been observed during physiological development in osteoclasts [14] and skeletal muscle cells [15] or after pathological viral infection [16]. In this process, cells fuse their nuclei and/or membranes, leading to the genesis of mononuclear or multinucleate cells, respectively.

Other mechanisms are directly associated with proliferative state of the cells.

**(1) Endoreduplication**

During this process, cells alternate S (DNA replication) and G phases, without performing mitosis and give rise to the genesis of autopolyploid cells (i.e., trophoblastic giant cells).

**(2) Endomitosis**

Cells can reach metaphase or anaphase A, but nuclear (karyokinesis) and cytoplasmic (cytokinesis) divisions are never observed; the best-studied example being polyploid megakaryocytes [17]. These cells enter mitosis but never fully separate sister chromatids or undergo cytokinesis, resulting in globulated polyploid nuclei [18, 19]. The regulatory mechanisms that control megakaryocytes polyploidization have been explored by different groups with a major focus on the regulation of mitotic phase and cytokinesis. Endomitosis appears to be due to a complex regulation of Cdk1/Cyclin B levels [20]. Studies of different megakaryoblastic cell lines suggest that endomitosis is promoted by the downregulation of Cyclin B/Cdk1 mitotic kinase activity [21, 22]; differently, in primary polyploid megakaryocytes, levels of cyclin B are reported to be upregulated [18, 23, 24]. Moreover, other studies have reported a reduction in the duration of the G1 phase correlated with overexpression of cyclin E [21, 25, 26]. Recent data have shown that cyclin E mediates its effect by promoting the expression of components of the prereplication complex (Cdc6 and MCM2). Overexpression of cyclin E can favor progression to S phase and cell cycling, thus promoting endomitosis and polyploidization of megakaryocytes [24].

**(3) Mitotic Slippage**

During this pathological process, cells present an altered Spindle-Assembly-Checkpoint (SAC). The SAC prolongs mitosis until all kinetochores are stably attached to spindle microtubules; when the SAC cannot be satisfied, cells exit mitosis without undergoing anaphase or cytokinesis (genesis of mononucleated tetraploid cells). Mitotic slippage has been observed for example in cells after prolonged mitotic arrest in response to spindle toxins [27] or in APC-deficient cells (adenomatous polyposis coli, gene frequently mutated in colon cancers) [28].

**(4) Incomplete Cytokinesis**

This process has been extensively described during pathological division and leads to the genesis of binucleated tetraploid cells. These cells can appear following dysfunction of any of a large number of different proteins controlling cytokinesis process [29]. In addition, bulk chromatin or even a single lagging chromosome trapped in the cleavage furrow can induce cytokinesis failure and tetraploidization [30, 31]. Remarkably, recent studies demonstrated that cytokinesis failure process is also a programmed step in normal development (as example: liver and heart tissues; see Section 3.1 for more details) producing differentiated binucleated tetraploid progenies [32–35]. Finally, it is important to note that whatever the mechanism of polyploidization, the increase in cellular DNA content will be associated with centrosomes amplification, which in certain cases could lead to the genesis of aneuploid progenies and CIN (see [13, 36] for reviews).

## 2. Hepatocytes Polyploidy and Liver Growth

### 2.1. Postnatal Development

**Figure 1:**
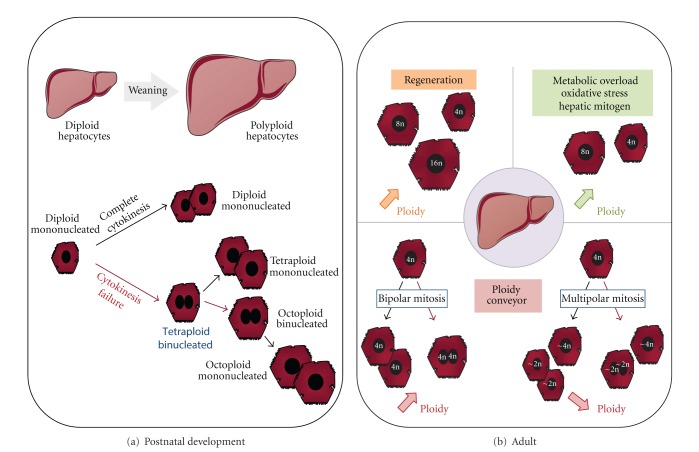
Hepatocytes polyploidization during development and in challenging circumstances: (A) polyploidization during postnatal liver growth. Hepatocytes in newborn are exclusively diploid (mononucleated 2n). At the weaning period, diploid hepatocytes can engage either into normal cell division cycle (black arrow) giving rise to two diploid hepatocytes or follow an adaptive cell cycle with cytokinesis failure (red arrow) giving rise to one binucleated tetraploid hepatocyte. By this process, progressive polyploidization takes place in the liver parenchyma and tetraploid and octoploid cell classes with one or two nuclei are formed. (B) Ploidy modification during physiopathological processes in adult liver. In adult, liver modulates its ploidy in response to different signals. Liver regeneration induced by partial hepatectomy leads to the disappearance of binucleated hepatocytes and the formation of mononucleated tetraploid and octoploid hepatocytes or even 16n contingent. DNA synthesis induced by chemicals or following oxidative damage and metabolic overload (copper/iron) is associated with a pronounced increase in the proportion of polyploid hepatocytes. Furthermore, in response to different unknown signals, hepatocytes can both increase (bipolar mitosis followed by cytokinesis failure) or decrease their ploidy (multipolar mitosis). In that case, near-diploid/near-polyploid contingents will be generated, leading to the genesis of genetically distinct daughter cells; black arrow: complete cytokinesis, red arrow: cytokinesis failure.

Hepatic polyploidy is a characteristic feature of mammalian liver and accompanies late fetal development and postnatal maturation [7, 37]. In rodents, through 14th embryonic development day (e.g., E14), most hepatoblasts are bipotent with the ability to differentiate into hepatocytes or into biliary cells; by E15 most hepatoblasts are committed to the hepatocyte lineage [38, 39]. During the remaining period of gestation and the first four postnatal weeks, hepatoblasts acquire functions of differentiated hepatocytes, and this period is correlated with a severe decline in proliferative state [40, 41]. During previous studies, we have observed that the liver is almost exclusively made up of diploid hepatocytes for the first three weeks after birth. After weaning (day 21), the proportion of diploid hepatocytes started to fall significantly, with the successive appearance of binucleated tetraploid (2 × 2n) and mononucleated tetraploid (4n) hepatocytes [32, 33] (see Figure 1). The hepatocyte ploidy level effectively reaches a plateau at maturity, octoploid (binucleated 2 × 4n and mononucleated 8n) hepatocytes appearing in significant numbers during the second and third months after birth [42]. Interestingly, a second wave of high ploidization has been also observed at senescence in different species [43]. For example, in humans, polyploid hepatocytes begin to appear during postnatal liver development; their accumulation rate stays stable during the maturity period, and finally a significant increase of polyploid cells is observed during ageing process [44]. In adults, 70% of all hepatocytes in rodents and 40% in humans are tetraploid [42, 45]. It has to be noticed that a negative correlation exists between mitotic index in the liver and the level of hepatocyte polyploidization found in different species [46, 47]. As example, mouse liver has a much lower mitotic index than rat liver and accordingly the highest level of hepatocytes polyploidization was found in the mouse liver.

### 2.2. Adult Liver

Interestingly, in the adult liver, the genesis of polyploid cells can be reinduced following a variety of signals (see Figure 1).

Indeed, after two-thirds hepatectomy, mitogenic signals (cytokines and growth factors; for review, see [48]) induce exit of quiescence (“priming”) of hepatocytes. These hepatocytes undergo one or two rounds of one or two rounds of cell division to restore the hepatic liver mass and this process is associated with a pronounced increase of polyploid hepatocytes [37, 46, 49, 50]. Several reports indicated that liver regeneration depends mainly on the proliferation of hepatocytes [48, 51]. However, it has to be noticed that hypertrophy of hepatocytes in the regenerated liver has also been described [52–54]. A recent study has conciliated with these two pathways by revealing that hypertrophy precedes proliferation in the regenerating liver [55]. Furthermore, this work also established that preexisting diploid and tetraploid mononucleated hepatocytes generate cells with tetraploid and octoploid nuclei, respectively, by an unconventional cell cycle, probably by endoreplication as hepatocytes entering into S phase and skipping mitosis. Finally, they also described that binuclear hepatocytes undergo reductive divisions to generate two mononuclear daughter hepatocytes of higher ploidy. All these processes lead to an increase in both size and ploidy of hepatocytes during liver regeneration. It is interesting to note, that Sigal and Coll have also observed that 16n populations are found in the hepatic tissue, during the second day of liver regeneration. At the end of the regenerative process, 16n contingent is no more present in liver parenchyma. The authors suggested that the disappearance of these highly polyploid hepatocytes is associated with the establishment of apoptotic mechanisms that target preferentially hepatocytes of advanced ploidy [37].

In adult rodents, DNA synthesis induced by chemical compounds is associated with modifications of polyploid status in the liver. For example, lead nitrate induces the genesis of binucleated hepatocytes [56]. Adjunction of hepatic mitogens such as sodium phenobarbitone [57], 1, 4-dichlorobenzene [58], or peroxisome proliferators [59] are known to favor octoploid mononucleated hepatocytes genesis.

Hepatic polyploidy can be also modified by pathological overload that induce liver lesions. Different studies have described that liver of Long-Evans Cinnamon (LEC) rat (animal model of human Wilson's disease), which exhibits abnormal hepatic copper and iron concentration due to the deletion of the p-type copper transport ATPase gene (Atp7b), possesses a feature of increase in polyploidy (enlarged hepatocytes with huge nuclei) and a delay in mitotic progression. Interestingly, injection of irondextran in normal mice induces liver polyploidization; this effect is inhibited by the oral intake of iron chelator [60, 61].

Hepatocytes submitted to oxidative stressors develop pronounced increase in their polyploid status. Gorla et al. have demonstrated that subsequent to radiation, hepatocytes exhibit evidence for oxidative injury with deletion of intracellular antioxidants (as glutathione and catalase) and for increase of polyploidy [62]. Furthermore, a study on rats indicates that the rate of reactive oxygen species generation exceeds the induced antioxidant ability with aging, generating a situation that favors oxidative stress and peroxidation. This state is correlated with changes in the proliferative potential of hepatocytes and an increase in the genesis of octoploid contingent [63]. Further evidence for the role of oxidative injury in polyploidy is provided by studies showing that in transgenic mice overexpressing copper-zinc-superoxide dismutase and glutathione peroxidase, which are antioxidants, PH-induced hepatic polyploidization is decreased [64]. Similarly, treatment with aminoguanidine, which attenuates oxidative stress, decreased polyploidy [65]. It is interesting to note that in others polyploid cell types, such as VSMC, a crucial role of oxidative stress in polyploidization process has been underlined. Indeed, McCrann and Coll have described that increased expression of an ROS-producing enzyme, Nox4 (member of the NADPH oxidase family) results in VSMC polyploidy [66]. A role of Nox proteins in megakaryocytes endomitosis has been also suggested. Treatment of mouse bone-marrow cultures with Nox inhibitors resulted in accumulation of MKs with low DNA content levels and significant reduction of higher ploidy MKs. Further examination indicated that Nox-inhibited MKs showed a notable decrease in the level of the G1 phase cyclin E, a cyclin associated with MK polyploidy, and its upregulation restored most of the effect of Nox inhibitors [67].

All together, these results underline an extensive correlation between the generation of polyploid hepatocytes and a variety of cellular stress in the adult liver; however, cellular and molecular mechanisms involved in ploidization modification during pathological state are not well characterized.

## 3. Signalling and Mechanism Controlling Physiological Hepatocytes Polyploidy 

### 3.1. Cellular Mechanism

Our team has focused on the understanding of polyploidy hepatocytes lineage. We previously unveiled that during postnatal development and more precisely after weaning, diploid hepatocytes (mononucleated 2N) can engage either into a normal cell cycle and give rise to two diploid hepatocytes or follow an adaptive cell cycle with incomplete cytokinesis and give rise to one tetraploid hepatocyte (binucleated 2 × 2n) [32, 33]. In these hepatocytes, karyogenesis is achieved but these cells are not able to establish the cleavage plane. Several studies have revealed that RhoA GTPase is a key player to ensure a successful cytokinesis, by regulating the organization of the actin cytoskeleton and myosin II activity at the cleavage plane [68, 69]. We revealed that in hepatocytes, deficiencies in cytoskeleton reorganization inhibit Rho-A GTPase recruitment to the cleavage plane; consequently the cytokinesis ring is never formed [32]. The genesis of such binucleated tetraploid cells is the crucial step for the establishment of gradual polyploidization during postnatal liver growth. Indeed these cells are capable to proliferate and to give rise to two mononucleated 4n cells, which, if they divide, can generate 2 × 4n binucleated or 8n mononucleated hepatocytes. 

In the heart, incomplete cytokinesis has been also implicated in the genesis of binucleated tetraploid cardiomyocytes. In mammals, the growth of embryonic heart results in proliferation of cardiomyocytes (hyperplasia) [70]. After birth, ventricular cardiomyocytes respond to an amplification of blood flow by an adaptive increase in volume (hypertrophy). This transition from hyperplasia to hypertrophy is correlated to a tetraploidization process [35, 71]. In this system, a drastic reduction of RhoA and its effector ROCK after birth could account for defects in the process of cytokinesis [72]. Indeed, in some diploid cardiomyocytes, a cytokinetic ring is formed but as it is not at all functional, cytokinesis is never achieved, and tetraploid binucleated cell is consequently generated [35]. Differently from hepatocytes, adult 4N cardiomyocytes are in a postmitotic state and display a low proliferative potential (for review, see [73]). Recently, Gao et al. have shown that RhoA regulation is also a key target in MKs polyploidization and differentiation. Indeed, they have demonstrated that downregulation of the guanine exchange factor ECT2 prevents RhoA activation and cleavage furrow ingression during endomitosis cycle, allowing the formation of ≥4N MKs [74].

Interestingly, even if failed cytokinesis is the major event for liver polyploidization during postnatal development, some studies reveal that under certain circumstances, cell fusion can also contribute to this process. Experiments on stem cells and therapeutic applications have discovered that polyploid hepatocytes can be generated following cell fusion between exogenous bone marrow cells and mature hepatocytes [75, 76]. Furthermore, Faggioli and Coll have shown that in adult liver, genesis of binucleated hepatocyte could be directly promoted by homotypic fusion but with a rare occurrence [77].

### 3.2. Molecular Mechanism

Recently, we discovered that the suckling-to-weaning transition strictly controls the establishment of the cytokinesis failure process in the liver. Using a specific immunocytochemistry approach to detect mitotic events in liver tissue, we showed that cytokinesis failure events never occurred in 19-day-old suckling rat (<3%); whereas in 19-day-old rats weaned early (at 15 days), such events were frequent (>35%), and numerous binucleated tetraploid hepatocytes were generated. It is interesting to note that while suckling is prolonged to 25 days, hepatocytes mostly enter into complete cytokinesis events (>95%—data not published). Moreover, we reported there was a new wave of proliferation in the liver associated with the establishment of these specific adaptive cell cycles [41, 78]. We pointed out that insulin signalling triggers incomplete cytokinesis cell cycle program. If the physiological rise in insulin after weaning was inhibited in rats (by destroying pancreatic beta cells with streptozotocin drug), hepatocytes did not undergo cytokinesis failure, whereas if this rise was further accentuated (by injecting insulin), cytokinesis failure was even more frequent and an increase in the genesis of binucleated tetraploid hepatocytes was observed. By investigating how insulin controls polyploidy program, we discovered that PI3 K/Akt pathway (signaling pathway regulating cellular homeostasis through its role in regulation of apoptosis, cell growth, cell cycle, cytoskeleton organization and angiogenesis; see [79] for, review) is a key regulator of cytokinesis through the control of cytoskeleton networks. Indeed, direct inhibition ok Akt by chemical compound (iAKT) in hepatocytes primary culture prevents the appearance of incomplete cytokinesis process. We also examined the cytoskeleton organization in treated cells; iAkt-treated cells that completed cytokinesis reorganized the actin cytoskeleton and recruited RhoA to the equatorial cortex by contrast to cells that did not complete cytokinesis. In the past, several studies in yeast, metazoans, and mammals underlined a role of insulin in the regulation of cell proliferation and growth, by controlling G1/S-and G2/M-specific checkpoints [80–82]. However, we have demonstrated for the first time in mammals that this hormone, through the PI3 K-Akt pathway, can also regulate late mitosis progression and tightly control physiological polyploidization process during liver development.

Interestingly, a role of PI3 K-Akt pathway has been also described during a pathological polyploidization process. By overexpressing Akt1, Hixon et al. have demonstrated that VSMCs are able to override the activity of the mitotic spindle checkpoint, facilitating unscheduled degradation of cyclin B, cell-cycle reentry (endoreduplication), and polyploidization process. The same results were obtained by incubating VSMCs isolated from normotensive animals with angiotensin II (regulator of hypertrophic signals during hypertension), which is a key activator of Akt1 in VSMCs. These results demonstrate that Akt1 regulates ploidy levels in VSMCs and contributes to vascular smooth muscle polyploidization and hypertrophy during hypertension [83].

## 4. Functions of Polyploid Hepatocytes

Many examples from the literature illustrate that the acquisition of a polyploid status confers specific biological properties of cells. In the yeast *Saccharomyces cerevisiae*, polyploidization alters the expression profile of specific genes and regulates certain aspects of physiology and cell morphology [84]. In plants, high polyploidy is correlated with epigenetic changes associated with hybrid vigor (stronger and taller plants) [85]. In mammals, polyploidization of megakaryocytes is associated with terminal differentiation and regulation of platelets formation and function [86]. Indeed, polyploidization process increases the overall MK mass, resulting in an increase in platelet formation. Furthermore, a study suggested that MKs from different ploidy levels produce platelets with different functions: platelets originating from high-ploidy MKs are thought to be more easily activated than platelets generated from MKs with a lower ploidy [87].

In the liver our understanding of the consequences for hepatocytes polyploidization still remains enigmatic. (1) Polyploidy could protect hepatocytes of genotoxic damage by increasing the number of copies of functional genes; this might be especially important for the liver that has a primary function in metabolizing and eliminating toxic compounds. (2) Polyploidy could be an economical solution to growth problems that occur when an organ work within its capabilities, avoiding the great demand in energy that represents cell division. (3) Finally, polyploidy could alter the expression profile of specific genes. Recently, two studies using multitest approach of modular biology underline alteration in a wide range of functional gene groups between diploid and polyploidy hepatocytes. The authors suggest a link between genome multiplication and emergence of specific pathways (increase in metabolic plasticity and for the protection of replicating DNA from oxidative damage) that could promote hepatocyte cell survival and tissue regeneration under stressful conditions [88, 89].

## 5. Perspectives

Cellular polyploidization is now well known to be correlated to chromosomal instability appearance and carcinogenesis process development. Indeed, in some tumor types, there is direct evidence for the development of aneuploidy from a transient 4n state [13]. However, the impact of polyploid hepatocytes status on hepatocarcinoma (HCC) is still in debate. Recently, Grompe and Coll have shown that hepatocytes can increase (failed cytokinesis) and reduce (multipolar mitosis) their ploidy, thus resulting in the concept of a “ploidy conveyor.” In their works, authors showed that this dynamic mechanism can induce the genesis of “near-diploid/polyploid”, that is, aneuploid hepatocytes in rodents and humans livers [90, 91]. Given the high tumoral potential of aneuploid cells in tissue, these data are quite surprising as spontaneous tumor in the liver is rarely observed. The genesis of such “near polyploid” cells could then finally represent a source of genetic diversity, providing a strong selective advantage in response to multiple environmental stressors, as it has been demonstrated in yeast. Further investigations on this topic promise to increase our understanding of the mechanisms and functional consequences of hepatocytes polyploidization and could offer insights into hepatic physiopathology.

## Abbreviations

APC: Adenomatous polyposis coli
CIN: Chromosomal instability
HCC: Hepatocarcinoma
iAKT: Inhibitor of AKT
LEC: Long-Evans Cinnamon
MK: Megakaryocyte
NOX: NADPH oxidase
PH: Posthepatectomy
PI3K: PI-3 kinase
SAC: Spindle assembly checkpoint
VSMC: Vascular smooth muscle cells.
